# Effects of Sulfamethazine and Cupric Ion on Treatment of Anaerobically Digested Swine Wastewater with Growing Duckweed

**DOI:** 10.3390/ijerph19041949

**Published:** 2022-02-10

**Authors:** Yu Xiao, Chunping Yang, Jay J. Cheng

**Affiliations:** 1 College of Environmental Science and Engineering, Hunan University and Key Laboratory of Environmental Biology and Pollution Control (Hunan University), Ministry of Education, Changsha 410082, China; xiaoyu302@hnu.edu.cn; 2 Guangdong Provincial Key Laboratory of Petrochemical Pollution Processes and Control, School of Environmental Science and Engineering, Guangdong University of Petrochemical Technology, Maoming 525000, China; 3 Maoming Engineering Research Center for Organic Pollution Control, Academy of Environmental and Resource Sciences, Guangdong University of Petrochemical Technology, Maoming 525000, China; 4 Department of Biological and Agricultural Engineering, North Carolina State University, Raleigh, NC 27695, USA

**Keywords:** swine wastewater, duckweed, heavy metal, antibiotic, sulfamethazine, cupric ion

## Abstract

Duckweed (*Spirodela polyrrhiza*) has the potential to treat anaerobically digested swine wastewater (ADSW), but the effects of antibiotics and heavy metals in ADSW on the treatment performance and mechanism of *Spirodela polyrrhiza* are not clear. Herein, an experiment was conducted to investigate the effects of sulfamethazine (SMZ) and cupric ion on NH4+-N and total phosphorus (TP) removal from synthetic ADSW. The activity of superoxide dismutase (SOD) and the contents of photosynthetic pigments, vitamin E, and proteins in duckweed were also evaluated. Under the stress of SMZ, duckweed showed excellent removal efficiency of nutrients, and the results of SOD activity and photosynthetic pigments content indicated that duckweed had good tolerance to SMZ. Interestingly, a combined application of SMZ and cupric ion would inhibit the nutrient removal by duckweed, but significantly increased the contents of photosynthetic pigments, proteins, and vitamin E. In addition, the consequence indicated that high value-added protein and vitamin E products could be produced and harvested by cultivating duckweed in ADSW. Furthermore, possible degradation pathways of SMZ in the duckweed system were proposed based on the analysis with LC-MS/MS. This research proposed a novel view for using duckweed system to remove nutrients from ADSW and produce value-added products under the stress of SMZ and cupric ion.

## 1. Introduction

Commercial feed additives are extensively used in livestock for the prevention of diseases. The feed additives often contain high levels of antibiotics and heavy metals [1]. Tetracycline, sulfamethazine (SMZ), and dihydrostreptomycin are commonly used for livestock to treat and prevent diarrhea rash and other diseases [2]. Heavy metals such as cupric and zinc ion are also used as feed additives to promote the development of many important organs and their physiological functions of livestock [3]. However, neither heavy metals nor antibiotics can be completely absorbed by livestock. About 50–95% of them are excreted in the animal urine and feces [4]. Therefore, high concentrations of heavy metals and antibiotics have been discovered in swine wastewater and even in anaerobically digested swine wastewater (ADSW) [5]. For example, the concentration of antibiotics in high-concentration wastewater in Jiangsu Province, China could reach 5.0 mg/L. Zinc and copper are also widespread in swine waste, with the concentration ranging from 0.1 to 0.2 mg/L, and have not exceeded comprehensive sewage discharge standards of 5 mg/L and 2 mg/L, respectively (China) [6].

Heavy metal pollution has attracted extensive attention because of its great damage to the human body and ecological environment, and heavy metals in wastewater are difficult to be removed by anaerobic digestion or phytoremediation [7]. Antibiotics are considered as “pseudo-persistent” pollutants because of their contamination to the ecosystem, including water, soil, and food [8,9]. A wide range of antibiotics has been discovered in groundwater, and their presence may promote antibiotic resistance of bacteria. Antibiotic and metal resistance genes has become one of the major public health problems [10].

Therefore, many efforts have been made to remove antibiotics and heavy metal pollution from wastewater, including the anaerobic digestion, advanced oxidation, photocatalysis, electrodialysis, and constructed wetlands [11]. However, the technologies have their limitations such as high cost or possible secondary pollution. Phytoremediation is an attractive approach because it is a plant-based biotechnology to extract, sequester, or detoxify pollutants [12]. Phytoremediation is widely used in the removal of nutrients in ADSW and has low-cost, wide applicability, and no secondary pollutants technology [13]. Moreover, in the process of phytoremediation, not only the nutrients in wastewater can be removed, but also the growing plants can be harvested to obtain value-added products, which is unmatched by chemical and physical methods [14]. Nutrients including phosphorus and nitrogen in ADSW could be absorbed by a macrophyte to produce starch, proteins, and other organic compounds, which could be used as value-added by-products such as biofuels and animal feeds [15,16].

Duckweed (*Spirodela polyrrhiza*) has attracted much attention in aquaculture wastewater treatment because of its remarkable advantages [17,18]. Duckweed has many advantages as shown below: (I) It is widely distributed in natural aquatic ecosystems and easy to harvest; (II) it can quickly absorb nutrients from wastewater and efficiently remove pollutants such as antibiotics, heavy metals, and endocrine disruptors [19]; (III) it can be used as livestock feed or processed into Chinese medicine; and (IV) it is rich in high value-added products such as proteins and starch. For example, Cheng et al. described that *Spirodela punctate 7776* could effectively remove nutrients from swine wastewater, with removal rates of approximately 0.13 mg·L^−1^·h^−1^ for PO_4_-P and 1.0 mg·L^−1^·h^−1^ for NH_4_^+^-N. It was found that the protein yield of duckweed was 10 times higher than that of soybeans [20].

There have been some studies about the effects of heavy metals and antibiotics on the removal of nutrients from ADSW by duckweed, while they mainly focus on the effects of single pollutants [21]. In fact, the co-contamination of multiple pollutants in wastewater is very common and the interaction among different pollutants cannot be ignored. The interaction between antibiotics and heavy metals has attracted increasing attention [22]. Antibiotics and heavy metals can form complexes that may alter the absorption, adsorption [23], photolysis, and bioavailability of the antibiotics and heavy metals [24]. The complexes are different from antibiotics and heavy metals themselves in terms of environmental risks. Literature has shown that the complexes are much more toxic to microorganisms and aquatic plants than the individual pollutant [25]. However, there is little information about the removal of combined pollution of antibiotics and heavy metals in ADSW.

In this study, the combined effects of different initial concentrations of SMZ and Cu^2+^ were evaluated in nutrient removal from synthetic ADSW with growing *Spirodela polyrrhiza*. At the same time, the growth conditions of the *Spirodela polyrrhiza* were investigated, including changes in superoxide dismutase (SOD) activity, photosynthetic pigment, vitamin E, and proteins content. In addition, the degradation pathway of SMZ in the *Spirodela polyrrhiza* system was investigated. This study provides novel information for phytoremediation of SMZ and cupric ion-contaminated ADSW with growing *Spirodela polyrrhiza* and better understanding of the role of SMZ and cupric ion in the phytoremediation system.

## 2. Materials and Methods

### 2.1. Chemicals

Sulfamethazine (SMZ or C_12_H_14_N_4_O_2_S; purity 99%) was purchased from Shanghai Yien Chemical Technology Co., Ltd. (Shanghai, China); Methyl alcohol and acetonitrile supplied by Tedia Company (Fairfield, IA, USA); and CuSO_4_·5H_2_O and other chemicals purchased from the Sinopharm Co. Ltd. (Shanghai, China).

### 2.2. Spirodela Polyrrhiza and Synthetic Wastewater

*Spirodela polyrrhiza* were collected from a fresh water pond, Hunan, China (113°09 S, 29°38 E). Before the experiment, duckweed was cultivated in buckets that contained diluted ADSW and put in an incubator at 25 °C. The light intensity was 75 μmol m^−^^2^ s^−^^1^, and the light period was 15:9 h for culture preparation. The wastewater was artificially synthesized with chemical reagents. The pH of the wastewater was adapted to 6.5 ± 0.5 via using 0.1 M NaOH. The synthetic ADSW was disinfected in an autoclave at 121 °C for 30 min before the experiment, and the ionic concentration of the substrate was listed in Table 1.

### 2.3. Identification of Duckweed

The DNA of duckweed was extracted using the Bacterial Genome Extraction Kit. The polymer chain reaction (PCR) primer 1 (F:5’ACTCGCACACACTCCCTTTCC-3’) and primer 2 (R:5’-GCTTTTATGGAAGCTTAAACAAT-3’) were used to amplify the primers from duckweed. The PCR product purification is operated in accordance with the standard operating procedure of magnetic bead purification. Then the purified PCR product was tested on the machine and the sequence similarity search was performed.

### 2.4. Experiment Design

The tests were implemented in beakers (500 mL) in an incubator. Each beaker had 200 mL of synthetic ADSW. The appropriate duckweed (0.5 g fresh weight) was selected and washed for the experiment. The nutrient level and growth status of duckweed was monitored by destructive sampling method. The experiments were carried out in triplicates. During the experiment, the pH was maintained at 6.2 ± 0.5 by adding 0.1 M NaOH or HCl.

To evaluate the combined effects of SMZ and cupric ion on nutrient removal, duckweed growth, and SMZ degradation pathway in duckweed on synthetic ADSW, different volumes of 0.2 g/L SMZ were added to imitate different concentrations of SMZ (0, 0.1, 0.5, 1, 2, and 5 mg/L); 3.93 g/L CuSO_4_·5H_2_O solution was added to simulate 1 mg/L Cu^2+^ concentration, which was used as the initial media for the experiment. Twelve × 3 groups of samples were used to conduct experiments: SMZ concentration ranged from 0.1 to 5.0 mg/L and copper ion concentration at 0 and 1.0 mg/L, and the experiments were performed in triplicate. Since SMZ and Cu^2+^ were very stable in the solution, SMZ and cupric ion were checked and replenished every 4 days.

### 2.5. Analytical Methods

An entire beaker was taken as a sample with destructive sampling to monitor the nutritional level. The strainer was used to harvest duckweed from each beaker. The harvested duckweed was wiped with filter paper until there was no water on the surface, and then the fresh weight of duckweed with electronic balance was quickly measured. The pH value of synthetic ADSW was measured with a pH meter (PHS-3C). NH_4_^+^-N, TP, Cu, and SMZ in wastewater were analyzed after duckweed harvest. The sample residual was filtered with a 0.45 μm Millipore filter. NH_4_^+^-N (HJ 535-2009) and TP (GB 11893-89) were determined as described by Hu et al. [18]. The Cu^2+^ concentration was determined by an atomic absorption spectrometer (AAS, Agilent, Santa Clara, CA, USA). The concentration of SMZ was measured with an HPLC (1260 Infinity II, Agilent, Santa Clara, CA, USA) equipped with UV-Vis detector. The mobile phase of SMZ was 65% ultrapure water (A) and 35% acetonitrile (B), the flow rate was 1 mL min^−1^, and the detector wavelength was 266 nm.

#### 2.5.1. Determination of Physical and Chemical Properties of Duckweed

The duckweed proteins contents were determined with a total proteins quantitative assay kit (Coomassie brilliant blue method, Nanjing Jiancheng Bioengineering Institute, Nanjing, Jiangsu, China). The carotenoids and chlorophyll a and b contents of the duckweed were detected in the absorbency of 470, 665, and 649 nm, respectively. The photosynthetic pigments were analyzed using the method described by Zhou et al. [26]. In brief, 0.1 g of freeze-dried duckweed was accurately weighed into a 100 mL saponification bottle, and 20 mL of absolute ethanol and 5 mL of 100 mg/L ascorbic acid was added. Then, 10 mL of potassium hydroxide (the mass fraction was 50%) was added. In a boiling water bath, condense reflux was done to complete the saponification. The saponification bottle was washed twice with anhydrous ether (100 mL), and the ether solution was poured into the separatory funnel together. After standing and layering for 5 min, ether extract was obtained and then evaporated to 2 mL by rotary evaporator in a 55 °C water bath. The remaining ether liquid was blow-dried with pure nitrogen, followed immediately by adding 2 mL of anhydrous ethanol as well, dissolving the extract, and centrifuging at 5000 r/min for 5 min; the content of vitamin E was analyzed by HPLC (chromatographic method: methanol: water = 98:2, column temperature 30 °C, wavelength 300 nm). To measure the vitamin E content in duckweed, some collected duckweed was freeze-dried overnight. Then saponification extraction was used to extract vitamin E, and its content in duckweed was determined with HPLC. The activity of superoxide dismutase (SOD) was detected using a total superoxide dismutase assay kit (Nanjing Jiancheng Bioengineering Institute, Nanjing, Jiangsu, China).

#### 2.5.2. Degradation Products Analysis

To study the possible degradation pathway of SMZ with duckweed, the possible degradation products of SMZ were measured with LC-MS/MS. An appropriate amount of duckweed was taken from the experimental group that contained SMZ concentration of 5 mg/L. First of all, the samples were cleaned by distilled water and freeze-dried to make a powder. Then the duckweed samples were ground to powder, and 20 mL of extract solution A (Methanol-Mcllvaine-0.01 mol·L^−1^ EDTA-2Na solution 1:1:1 volume ratio, pH adjusted to 3 by phosphoric acid) was added. The mixture was sonicated for 30 min, then centrifuged at 4000 r·min^−1^ for 20 min to extract the supernatant, and repeated twice. The supernatant was collected and rotary evaporated to 20 mL, and the remaining solution was extracted via solid phase extraction. Finally, the solution was filtered and analyzed with a LC-MS/MS (Agilent, Beijing, China). The eluents were mixed and analyzed by liquid chromatography-mass spectrometry coupled with ZORBAX Eclipse Plus C18 Rapid Resolution HD column (50 mm × 2.1 mm, 1.8 μm). A five microliter sample was injected into the HPLC for analysis. Ultrapure water (A) and acetonitrile (B) were used as the carrier at a flow rate of 0.20 mL min^−1^. The following gradient was used: 0–12 min, 10% B to 60% B; 12–14 min, 60% B to 95% B; 14–16 min, 95% to 10% B; 16–17 min, 10% B. The instrument parameter settings were described by Zhang et al. [27]. In addition, the concentration of SMZ was determined by HPLC (Agilent, Waldbronn, Germany), which was furnished a UV–vis detector. Kromasil C18 column (5 μm, 4.6 mm × 250 mm) was as the column at 30 °C. The mobile phase of SMZ was acetonitrile and 0.1% (*v*/*v*) of acetic acid aqueous solution (45:55, A/B), and the flow rate was 1 mL min^−1^; the detector was at a wavelength of 270 nm.

### 2.6. Statistical Analysis

All figures in this work were performed using Origin 2017 (Originlab, Northampton, MA, USA). The mean ± SE (standard error) of the three replicates used to describe the consequence, and the differences of data were evaluated by using SPSS 20 (SPSS, Chicago, IL, USA) with one-way ANOVA.

## 3. Result and Discussion

### 3.1. Spirodela Polyrhiza

**Figure 1:**
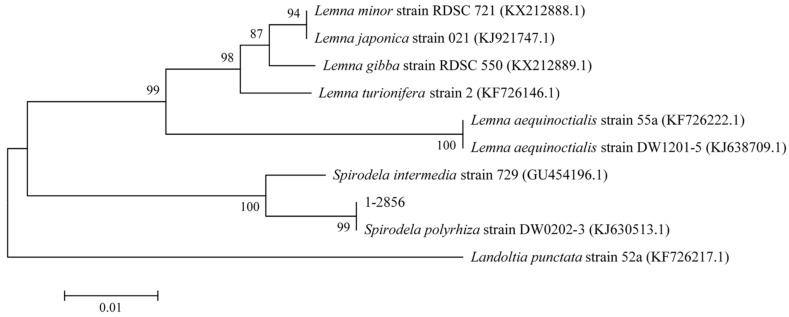
Tree of the tested duckweed on the basis of partial primer 1 sequences. Numbers in the parentheses are accession numbers of each sequence in GenBank Numbers at the nodes and indicate bootstrap values (expressed as a %) with 1000 replicates. The scale bar measures the distance between species.

The fronds of *Spirodela polyrhiza* are flat, the length of each frond is about 4–8 mm, the front surface is green, and the back surface is purple. According to the blast results, the sample (1-2856) has the closest match with chloroplast Spirodela polyrhiza, and the homology reached 99% similarity with *Spirodela polyrhiza* strain DW0202-3 (KJ630513.1). The phylogenetic analysis results of the duckweed are presented in Figure 1.

### 3.2. Nutrient Removal

**Figure 2:**
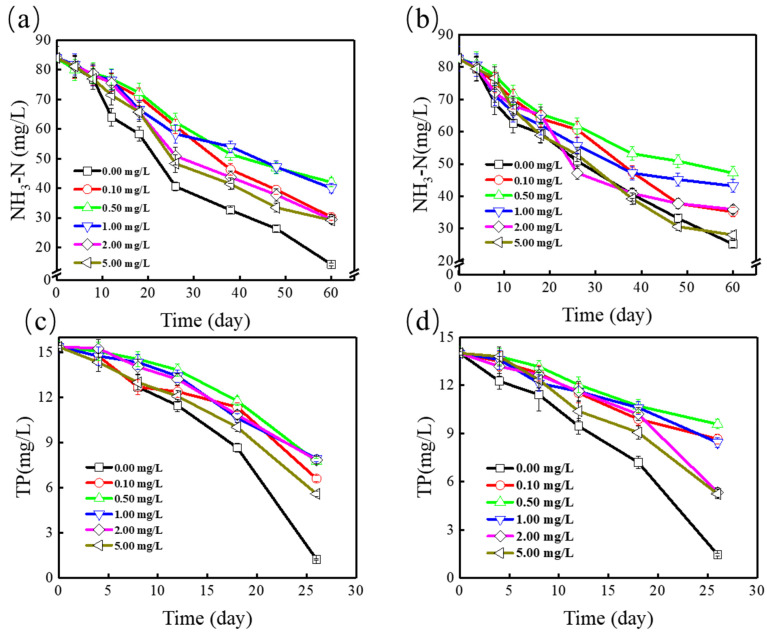
Nutrient ((**a**) NH_4_^+^-N and (**b**) TP) removal by *Spirodela polyrrhiza* at various concentrations of SMZ; Nutrient ((**c**) NH_4_^+^-N and (**d**) TP) removal by *Spirodela polyrrhiza* at various concentrations of SMZ and 1 mg/L Cu^2+^.

The influence of SMZ and Cu^2+^ on NH_4_^+^-N and Total phosphorus (TP) removal by *Spirodela polyrrhiza* within 60 days was illustrated in Figure 2. As shown in Figure 2a, in the control group, the NH_4_^+^-N concentration within 60 days decreased to 14.89 mg/L. There were clearly three phases for NH_4_^+^-N removal by *Spirodela polyrrhiza*. First, the duckweed was in a lag period, the NH_4_^+^-N removal efficiency was very low, only 0.778 mg·L^−1^·d^−1^, and then the rapid growth period of duckweed occurred and the NH_4_^+^-N removal rate reached 1.831 mg·L^−1^·d^−1^. When the TP was completely consumed, the removal efficiency dropped to 0.756 mg·L^−1^·d^−1^. The removal rate of NH_4_^+^-N increased speedily when the SMZ concentration increased from 0.1 to 5.0 mg/L, the values were 64.29%, 49.97%, 52.35%, 64.93%, and 65.27%, respectively (*p* < 0.05). The results showed that as the concentration of SMZ increased from 0.1 to 5.0 mg/L, the inhibitory effect on NH_4_^+^-N removal gradually decreased.

In addition, the effect of SMZ in the TP removal was observed in Figure 2b. The removal rate of TP gradually increased in the control group within 26 days, from 0.515 mg·L^−1^·d^−1^ to 0.929 mg·L^−1^·d^−1^. In the control group, the TP concentrations within 26 days declined to 1.24 mg/L, and the removal efficiency increased to 91.93%. The removal efficiency of TP increased slowly under the SMZ concentration that enhanced from 0.1 to 5.0 mg/L; the values were 44.52%, 45.81%, 43.88%, 50.97%, and 64.50%, respectively (*p* < 0.05). The results showed that the nutrient removal efficiency of duckweed was inhibited under the stress of SMZ, but the nutrient removal effect could reach more than 60%.

It has been reported that duckweed could reduce the damage from antibiotics by stimulating the secretion of biogenic amines [28]. Biogenic amines are generally low-molecular-weight organic compounds with biological activity. As a low-molecular-weight organic base, biogenic amines are commonly found in flora cells and play a significant role in eliminating excessive free radicals. Xiong et al. has also shown that SMZ is very toxic to microalgae, because it disrupts cell structure and organelles by interfering with the homeostasis of reactive oxygen species (ROS) regulation and energy transduction. A small quantity of ROS is beneficial to duckweed growth, which could be as a semaphore to cellular security response. However, the accumulation of ROS could result in peroxidation injury for duckweed growth [26].

There was insufficient SMZ to trigger the responsible biogenic amines and ROS at the lower SMZ concentration. However, SMZ will produce insufferable toxicity to nitrifying bacteria, which has a significant effect on removing ammonia nitrogen. Nitrifying bacteria play an important role in the purification process of nitrogen cycle water quality. When nitrifying bacteria decrease, ammonia content in water would increase, which was the main reason for SMZ inhibiting the removal of NH_4_^+^-N. On the contrary, as the concentration of SMZ increased, the amount of ROS and biogenic amines produced in duckweed increased significantly. ROS could act as a second messenger to initiate the defense response of the cell, causing superoxide dismutase; biogenic amine secretions were produced for maintaining the balance of the antioxidant system. They reduced the damage of SMZ for duckweed cells and rhizosphere microorganisms, thereby promoting *Spirodela polyrrhiza* to remove nutrients efficiency. These results were consistent with that reported by Xiong et al. [29]. In summary, SMZ at low concentration was toxic to *Spirodela polyrrhiza*, but as the concentration of SMZ increased, it promoted the removal of nutrients from synthetic ADSW by the duckweed.

The interaction of Cu^2+^ and SMZ on duckweed nutrient removal was observed in Figure 2c,d. With the combination of 1 mg/L Cu^2+^ and low concentration of SMZ (0.1–1.0 mg/L), an apparent synergistic effect was observed. The nutrient removal effect of duckweed was obviously inhibited (*p* < 0.05), and the nutrient removal rate of duckweed was significantly lower than that with SMZ alone. However, when the concentration of SMZ was high (2.0–5.0 mg/L), it had no obvious inhibitory effect on duckweed removal of nutrients. The combined effect of Cu^2+^ and SMZ has a greater inhibitory effect for duckweed removing nutrients than that with SMZ alone. This may be attributed to the fact that under the conditions of low concentrations of SMZ and Cu^2+^, copper could chelate the donor groups in SMZ to form a stronger complex, which was difficult to degrade by duckweed and has a harmful effect on rhizosphere microorganisms [6], thus inhibiting the removal of nutrients by duckweed from synthetic ADSW. In comparison, under the conditions of high concentrations of SMZ and Cu^2+^, the compound formed has a weaker affinity for duckweed roots and a faster dissipation rate. Therefore, the compound has less influence on rhizosphere microorganisms, thereby promoting the removal of nutrients by the duckweed.

### 3.3. Effects on Antioxidant System

The effects of SMZ on SOD activities in *Spirodela polyrrhiza* were studied to investigate the antioxidant responses of the duckweed. Compared with the control group, duckweed exposed to 0.1–5.0 mg/L SMZ resulted in a significant SOD activity increase within 8 days.

It has been reported that various environmental stresses could promote the formation of ROS. For example, heavy metals and antibiotics could encourage the formation of superoxide radicals (O^2−^), which could cause oxidative stress [30]. SOD could convert O_2_^−^ to H_2_O_2_, while catalase (CAT) could degrade H_2_O_2_ into H_2_O and O_2_ [31]. Our experimental results indicated that with the addition of SMZ, the activity of SOD changed dramatically. The activity of SOD increased first and then decreased after 26 days of exposure, which indicated that duckweed exposed to SMZ would interfere with the balance of normal redox state, and as a result, induce more SOD to deal with excess O_2_^−^. However, the decrease in SOD activity might indicate that duckweed was gradually adapting to the environment [32].

**Figure 3:**
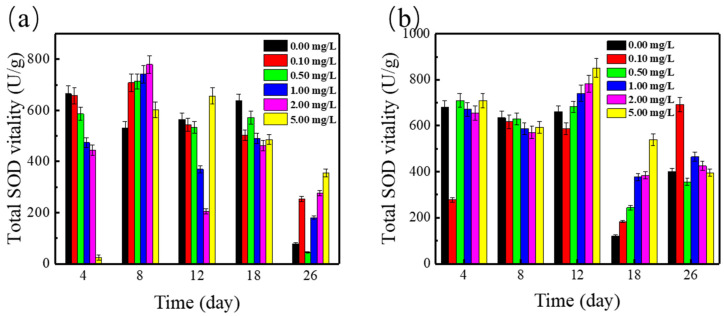
(**a**) Total SOD vitality in *Spirodela polyrrhiza* at various concentrations of SMZ; (**b**) Total SOD vitality in *Spirodela polyrrhiza* at various concentrations of SMZ and 1 mg/L Cu^2+^.

The interaction of Cu^2+^ and SMZ with SOD activities in duckweed is observed in Figure 3. Compared with SMZ only-treated duckweed, SOD activity did not remarkably (*p* < 0.05) increase after the duckweed was exposed to both SMZ and Cu^2+^ for 26 days. SOD activities also increased firstly and then declined after 26 days of exposure, but the highest SOD value was observed in the concentration of 5 mg/L SMZ treatment on the 12 days, correspondingly to 851.6 u/g. SOD could remove O_2_^−^ and protect cells from damage, while the presence of Cu^2+^ will promote the production of ROS and make SOD activity increase. With the increase of time, duckweed gradually adapting to the environment makes the SOD content gradually decline [26]. The results suggested that duckweed could quickly adapt to environmental stress and has good adaptability, making the *Spirodela polyrrhiza* system a promising choice for the treatment of ADSW polluted by SMZ and cupric ion.

### 3.4. Effects on Photosynthetic Pigments

**Figure 4:**
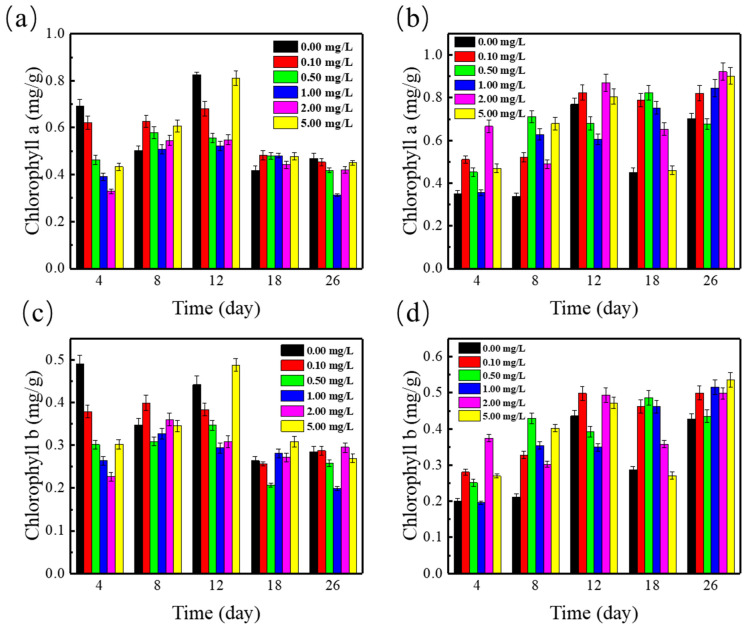
Photosynthetic pigments content ((**a**) chlorophyll a; (**b**) chlorophyll b in *Spirodela polyrrhiza* at various concentrations of SMZ. Photosynthetic pigments content ((**c**) chlorophyll a; (**d**) chlorophyll b) in *Spirodela polyrrhiza* at various concentrations of SMZ and 1 mg/L Cu^2+^.

The effects of SMZ alone on the change of photosynthetic pigments in *Spirodela polyrrhiza* were studied, and the results are shown in Figure 4. The content of chlorophyll a and b increased first and stabilized gradually. The content of chlorophyll a and b was inhibited compare to the control. In addition, with the increase of SMZ concentration, the content of chlorophyll a and b was inhibited more obviously (*p* < 0.05). A similar result was reported by Xu et al. who found that tetracycline decreased photosynthetic pigments contents in wheat seedlings significantly [33]. Furthermore, on the 12 days of the culture period, the maximum photosynthetic pigments appeared at the 5 mg/L SMZ. The maximum value of chlorophyll a and b was 0.81 mg/g and 0.49 mg/g, respectively. When duckweed was exposed to SMZ solution for the first time, perhaps due to its protective mechanism, photosynthetic pigments began to be synthesized in large quantities to deal with the accumulation of SMZ and reduce the toxicity of SMZ to the duckweed [34]. In addition, the reduction of photosynthetic pigments may be caused by SMZ inhibiting the synthesis of folic acid [35], and studies have shown that the biosynthesis of photosynthetic pigments is closely related to the state of folic acid [25]. In our experiments, the photosynthetic pigments content of duckweed was similar to the control group after 18 days. The results indicated that SMZ had little damage on the organelle membranes involved in photosynthesis and the leaves related in photosynthetic mechanism and function, thus maintaining the same photosynthetic efficiency level as the control. SMZ probably inhibited the synthesis of folic acid, thereby reducing the photosynthetic pigments in *Spirodela polyrrhiza.*

The interactions of Cu^2+^ and SMZ with the changes of photosynthetic pigments in *Spirodela polyrrhiza* were studied to evaluate the effect of these contaminants on the photosynthetic activity of duckweed. Compared with SMZ-contaminated duckweed ADSW treatment system, the photosynthetic pigment increased significantly in *Spirodela polyrrhiza* under the stress of SMZ and cupric ion. It has a significant antagonism effect on the content of chlorophyll a and b in *Spirodela polyrrhiza*, which could greatly increase (*p* < 0.05) the content of chlorophyll a and b in duckweed and has a greater impact on the content of chlorophyll a (maximum value of 0.922 mg/L). It is believed that chlorophyll a was mainly responsible for photosynthesis, while chlorophyll b can absorb energy and transfer it to chlorophyll a [36]. The increase in chlorophyll a and b content may have been to cope with the accumulation of SMZ and Cu^2+^ in duckweed and reduce the toxicity of SMZ and Cu^2+^ to the duckweed, so a large amount of photosynthetic pigments was synthesized. However, the increase of chlorophyll a and b content in varying degrees may indicate that duckweed under the stress of SMZ and copper ions caused the imbalance of photosynthetic activity in duckweed [37].

### 3.5. Effects on Proteins and Vitamin E

**Figure 5:**
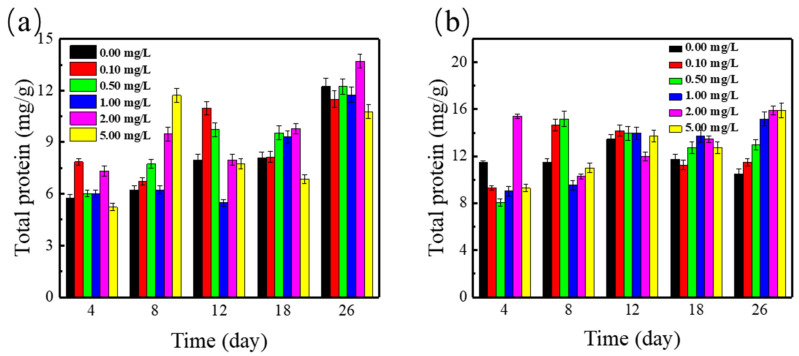
Proteins content in *Spirodela polyrrhiza* under (**a**) various concentrations of SMZ and (**b**) various concentrations of SMZ and 1 mg/L Cu^2+^.

Proteins could produce by *Spirodela polyrrhiza* removal of nutrients to fulfill growth needs [38]. The stress of induced xenobiotics could trigger proteins metabolism in plants, which may be part of the plant’s defense system against exogenous secretions [39]. The single effect of SMZ on the contents of proteins in *Spirodela polyrrhiza* has been studied, and the results are as shown in Figure 5; therefore, duckweed could absorb part of the NH_4_^+^-N synthetic protein, so the protein content increases with the progress of the experiment. Compared with the control group, after exposure to 0.1–5.0 mg/L SMZ, the duckweed proteins content increased significantly (*p* < 0.05) within 26 days. So as to decrease the injure from SMZ, *Spirodela polyrrhiza* synthesizes proteins by providing amino acids to promote better duckweed growth. Thus, the proteins of *Spirodela polyrrhiza* enhanced swiftly, which clearly (*p* < 0.05) exceeded the control.

The interaction of Cu^2+^ and SMZ with proteins content in duckweed was noticed in Figure 5b. Compared with the single application of SMZ to the duckweed system, a major increase of proteins content was observed in the duckweed under the combined effects of Cu^2+^and SMZ. This phenomenon indicated that a synergy effect was found in the combination of Cu^2+^and SMZ for the synthesis of proteins in duckweed, which could promote the production and accumulation of proteins in duckweed.

Vitamin E has a strong antioxidant activity, which could affect the biological growth through different immune regulation mechanisms and prevent the occurrence of infection. Vitamin E contains both tocopherols and tocotrienols, as well as lipid-soluble antioxidants that can regulate lipid peroxidation. Furthermore, α-tocopherol not only scavenges free radicals by triggering motion in the lipid bilayer, but may also help in fine-tuning the delivery of specific signals outside the chloroplast. Vitamin E was essential for plant development and helps to provide the appropriate response to a variety of environmental stresses [40].

**Figure 6:**
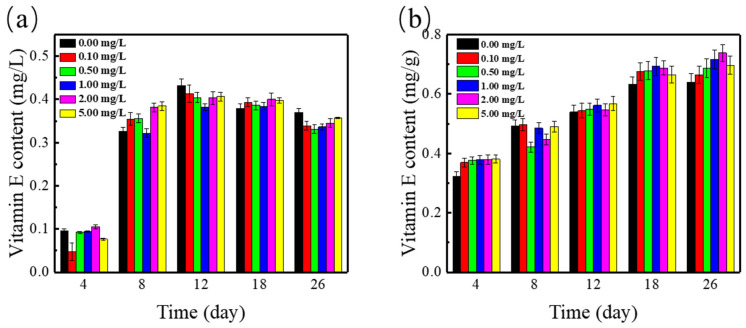
Vitamin E content in *Spirodela polyrrhiza* under (**a**) various concentrations of SMZ and (**b**) various concentrations of SMZ and 1 mg/L Cu^2+^.

The individual effect of SMZ on the contents of vitamin E in *Spirodela polyrrhiza* was studied in Figure 6. Within the 26 days, the content of vitamin E gradually enhanced (*p* < 0.05) and remained stable. The Vitamin E content was the highest in the concentration of 5 mg/L SMZ. When the duckweed was exposed to ADSW for the first time, SMZ and high-concentration nutrients added stress to duckweed, which resulted in a gradual increase in vitamin E. Duckweed gradually adapted to the environment and vitamin E maintained a relatively stable value, which indicated that duckweed has a high tolerance and adaptability to pollution. However, the stress mechanism of SMZ on duckweed always exists, so the vitamin E content does not drop to zero at a concentration of 0.1–5 mg/L SMZ, but maintains at a stable value.

The interaction of Cu^2+^ and SMZ with the changes of vitamin E contents in *Spirodela polyrrhiza* was studied, and the results are indicated in Figure 6b. The Vitamin E content in duckweed under the combined effect of Cu^2+^ and SMZ increased significantly in synthetic ADSW. As the culture time increased, the vitamin E content also increased in duckweed. The Vitamin E content was the highest under the combination of 2 mg/L SMZ and Cu^2+^. Vitamin E could diametrically restore ROS and participate in the clearing of ROS as a substrate for the enzyme [41]. Vitamin E was very important to the growth of plants and helped to provide the most suitable response to a variety of environmental stresses. Therefore, vitamin E has also been proven to be a valid marker of plants contamination [40]. High content of vitamin E in *Spirodela polyrrhiza* indicated that *Spirodela polyrrhiza* was under severe pollution stress. The higher vitamin E content was related to the synergistic effect of compound pollution, which indicated that the compound pollution of SMZ and Cu^2+^ may provide a prospective solution to enhance the accumulation of vitamin E in *Spirodela polyrrhiza.*

### 3.6. Degradation Products and Degradation Pathway of SMZ Analysis

**Figure 7:**
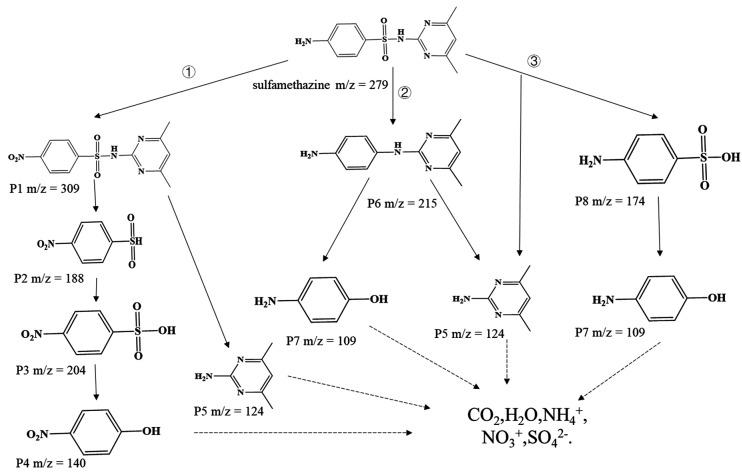
Possible pathways for biodegradation of sulfamethazine (SMZ).

At present, the degradation pathways of SMZ in duckweed system mainly include photodegradation, volatilization, biosorption, and biodegradation [4]. Biosorption and biodegradation are primary pathways for the removal of antibiotics from synthetic ADSW [14]. At the same time, the growth of a large area of duckweed also hinders the penetration of sunlight, so the removal of SMZ through photodegradation could also be ignored. During the biodegradation of SMZ, eight intermediates were detected based on LC-MS/MS analysis. To further understand the biodegradation process of SMZ in the *Spirodela polyrrhiza* system, three possible degradation pathways were proposed in Figure 7 based on the detected intermediates and results reported in previous studies. Pathway 1 was mainly the process of hydroxylation and removal of SO_2_^.^ P1 was obtained by the oxidation of the aniline moiety of SMZ [42]. The product P2 was generated by oxidation of the amino group on the benzene ring of P1. Then, P3 was obtained by hydroxylation of sulfonyl group on P2. The formed P3 became 4-nitrophenol (P4), with removal of sulfur dioxide [43,44]. Pathway 2 was chiefly the separation of sulfonyl groups. SMZ lost a sulfonyl group and the C-N bond broke to form P5 and P7 [45]. Pathway 3 commonly includes the scission of S-N bonds. The S-N bond was cleaved to constitute P5 and P8, and next P8 was changed to P7 by oxidation reaction [27]. In the end, the formed degradation products P4, P5, and P7 can be further mineralized into small molecules, such as water and carbon dioxide [46].

## 4. Conclusions

SMZ and cupric ion-polluted ADSW were treated by *Spirodela polyrrhiza*. With the combination of Cu^2+^ and a low concentration of SMZ (0.1–2 mg/L), an apparent synergistic effect was observed and the nutrient removal efficiency of duckweed was significantly inhibited. When SMZ was used alone, the SOD value first increased and then decreased to a stable value, indicating that duckweed has a good tolerance to SMZ pollution. The same performance appeared in the combined use of SMZ and cupric ion, but the adaptation time became longer, indicating that duckweed was also highly resistant to the combined pollution of SMZ and cupric ion. The single effect of SMZ on the photosynthetic pigments in duckweed was studied, and the content of chlorophyll a and b was significantly inhibited, while the combined SMZ and cupric ion had an antagonistic effect to enhance the photosynthetic pigments. The contents of proteins and Vitamin E in duckweed increased gradually with the addition of single SMZ. The combination of SMZ and cupric ion had a synergistic effect, which resulted in a significant increase of protein and VE contents in duckweed. These results indicated that duckweed has the potential to produce high value-added proteins and vitamin E in the treatment of ADSW containing high concentrations of SMZ and cupric ion. Three possible degradation pathways were proposed according to the consequences from LC-MS/MS analysis.

## Figures and Tables

**Table 1:** Ionic concentrations of synthetic ADSW.

Ionic Concentrations (mg/L)	Value
COD	220.05 ± 11.36
NH_3_-N	82.84 ± 3.47
PO_4_-P	15.10 ± 0.97
NO_3_-N	100.57 ± 2.59
Ca^2+^	119.65 ± 2.15
Mg^2+^	25.16 ± 2.32
K^+^	98.97 ± 4.31
Na^+^	175.58 ± 1.86
Cl^−^	280.43 ± 10.34
SO_4_^2−^	125.82 ± 6.73
Fe-EDTA	40.15 ± 2.43
Minor elements	2.61 ± 0.18

## Data Availability

Not applicable.
